# Eleven remarkable Diptera species, emerged from fallen aspens in Kivach Nature Reserve, Russian Karelia

**DOI:** 10.3897/BDJ.6.e22175

**Published:** 2018-02-05

**Authors:** Alexei Polevoi, Anna Ruokolainen, Ekaterina Shorohova

**Affiliations:** 1 Forest Research Institute, Petrozavodsk, Russia

**Keywords:** Diptera, Russia, dead wood, aspen, wood-decaying macrofungi

## Abstract

**Background:**

In 2016, saproxylic Diptera associated with aspen (*Populus tremula* L.) logs were studied in the Kivach Nature Reserve, Russian Karelia, using trunk emergence traps.

**New information:**

Eleven rare species of Diptera (families Limoniidae, Scatopsidae, Axymyiidae, Mycetophilidae, Sciaridae, Platypezidae, Syrphidae and Clusiidae) with poorly known distribution and ecology were recorded. For each species, basic diagnostic characteristics were provided along with the information on microhabitats. An attempt was also undertaken to outline possible associations with wood-decaying macrofungi using nonparametric correlation.

## Introduction

Saproxylic Diptera have never been specially studied in Russian Karelia, except for certain groups partly associated with wood-decaying fungi (Yakovlev 1988, Yakovlev 1993, Yakovlev 1995, Jakovlev 2011). Only a few works provide information on abundance and microhabitats for some rare or red-listed species associated with dead wood (Humala and Polevoi 2015, Siitonen et al. 1996, Polevoi and Salmela 2014, Yakovlev et al. 2000). In 2015, research which aimed to study the insect fauna on fallen logs of different tree species, was started. As a result, a number of remarkable species of Coleoptera, Hymenoptera and Diptera were recorded (Polevoi and Pilipenko 2016, Polevoi et al. 2017). In 2016, trapping was continued concentrating on fallen aspens with different degrees of decomposition. In total, 105 species of Diptera were collected, including a range of poorly known taxa Suppl. material 1. Here the most remarkable findings are presented.

## Materials and methods

The trapping was undertaken in the Kivach Nature Reserve, Republic of Karelia. The biotope was *Oxalis acetosella*-*Vaccinium myrtillus* type spruce forest, over 150 years old (for details see Shorohova et al. 2016). Trunk emergence traps (Polevoi and Pilipenko 2016) were used as a collecting method. Fourteen traps were set on the fallen aspens, which had died from 1 to 16 years ago. The time since death was determined using dendrochronological methods (Shorohova et al. 2016). The traps were installed on 25 April 2016 and kept until 28 September 2016, with monthly checking. Diameter at the breast height, decay class (Shorohova et al. 2016) and the tree mortality mode (windthrow or windbreak) were recorded for each tree. The species composition of wood-decaying macromycetes was identified as well. The inventory of the fruitbodies was conducted in May, June, August and October, 2015 as well as in July and September, 2016. The presence-absence was recorded for all species on each log, counting one or several fruitbodies of a particular species on an individual dead tree as one occurrence. Remarkable Diptera species were recorded in 10 of the 14 installed traps (Table 1).

Series of images were captured with Leica MZ 9.5 and Leica DM1000 stereomicroscopes supplied with Leica DFC290 and LOMO MC-6.3 digital cameras. Images were then z-stacked using Helicon Focus software (http://www.heliconsoft.com/heliconsoft-products/helicon-focus).

Association with fungi was analysed using Pearson's non-parametric rank-order correlation coefficient (RS) calculated with PAST software (Hammer et al. 2001).

Insect and fungal specimens are stored in the collection and herbarium of the Forest Research Institute, Petrozavodsk (FRIP).

## Taxon treatments

### Gnophomyia acheron

Alexander, 1950

http://www.catalogueoflife.org/annual-checklist/2017/details/species/id/b9c29484b800e7dffeeced4fa3b973bc

#### Materials

- **Type status:** Other material. **Occurrence:** recordedBy: A. Polevoi; sex: 7 males, 2 females; **Location:** country: Russia; stateProvince: Karelia; locality: Kivach Nature Reserve; verbatimLatitude: 62.281; verbatimLongitude: 33.967; verbatimCoordinateSystem: Decimal degrees; verbatimSRS: WGS84; **Identification:** identifiedBy: A. Polevoi; **Event:** samplingProtocol: Trunk emergence trap; eventDate: 2016-05-26/06-2; **Record Level:** institutionCode: FRIP

#### Diagnosis

Medium sized dark species, distinguished from other *Gnophomyia* by the structure of male and female genitalia (Fig. 1; see also Hancock (2008), fig. 6, 10).

#### Distribution

Palaearctic. Only recently discovered in Finland and Russian Karelia (Polevoi and Salmela 2014, Starý and Salmela 2004).

#### Ecology

Saproxylic species, associated with different deciduous trees (Krivosheina 2008). In Finland and Karelia, so far collected exclusively on aspen (Hancock 2008, Halme et al. 2012). According to the authors' data, this species prefers huge wind-broken aspens with a diameter over 70 cm, fallen 7 years ago (Table 1). It favoured the trunks colonised by *Lentaria afflata* (RS=0.56, P<0.05) and *Subulicystidium longisporum* (RS=0.62, P<0.05), but seemed to avoid those decayed by *Phellinus tremulae* (RS=-0.56, P<0.05).

#### Conservation

Red-listed in Finland, category VU (Penttinen et al. 2010).

### Atypophthalmus (Microlimonia) machidai

(Alexander, 1921)

http://www.catalogueoflife.org/annual-checklist/2017/details/species/id/d9bb1dfccd0cc6dea751bed18d0696c0

#### Materials

- **Type status:** Other material. **Occurrence:** recordedBy: A. Polevoi; sex: 1 male, 1 female; **Location:** country: Russia; stateProvince: Karelia; locality: Kivach Nature Reserve; verbatimLatitude: 62.281; verbatimLongitude: 33.967; verbatimCoordinateSystem: Decimal degrees; verbatimSRS: WGS84; **Identification:** identifiedBy: A. Polevoi; **Event:** samplingProtocol: Trunk emergence trap; eventDate: 2016-06-23/07-21; **Record Level:** institutionCode: FRIP

#### Diagnosis

Medium sized species with peculiar male genitalia (Fig. 2), see also (Podenas and Gelhaus 2007, fig. 51)

#### Distribution

Palaearctic and Oriental regions (Oosterbroek 2017). Karelian record is the northernmost one. Closest locations are in Poland, Lithuania and Moscow province in Russia.

#### Ecology

Saproxylic species. Larvae develop under the bark of various trees (Krivosheina 2010). This species was collected from a wind-broken aspen, colonised by 13 species of macrofungi (Table 1).

### Discobola parvispinula

(Alexander, 1947)

http://www.catalogueoflife.org/annual-checklist/2017/details/species/id/8bb2ed786a6d52e53c05e998f1957b9b

#### Materials

- **Type status:** Other material. **Occurrence:** recordedBy: A. Polevoi; sex: 1 male; **Location:** country: Russia; stateProvince: Karelia; locality: Kivach Nature Reserve; verbatimLatitude: 62.281; verbatimLongitude: 33.967; verbatimCoordinateSystem: Decimal degrees; verbatimSRS: WGS84; **Identification:** identifiedBy: A. Polevoi; **Event:** samplingProtocol: Trunk emergence trap; eventDate: 2016-07-21/08-19; **Record Level:** institutionCode: FRIP

#### Diagnosis

Distinguished from other *Discobola* by more heavily marked wings and the structure of the male genitalia (Fig. 3), see also (Starý 1974, fig. 5, 6) .

#### Distribution

Palaearctic (Oosterbroek 2017). Karelian record is the northernmost one. Closest locations are in Poland, Lithuania and the Republic of Bashkortostan in Russia.

#### Ecology

Poorly known, though associations with dead wood and wood-destroying fungi were reported (Ševčík 2006, Wiedenska 2007). This species was collected from a windthrown aspen, colonised by 15 species of macrofungi (Table 1).

### Limonia badia

(Walker, 1848)

http://www.catalogueoflife.org/annual-checklist/2017/details/species/id/62f9e86b98f925526e31a0a3ac4c380a

#### Materials

- **Type status:** Other material. **Occurrence:** recordedBy: A. Polevoi; sex: 31 males, 13 females; **Location:** country: Russia; stateProvince: Karelia; locality: Kivach Nature Reserve; verbatimLatitude: 62.281; verbatimLongitude: 33.967; verbatimCoordinateSystem: Decimal degrees; verbatimSRS: WGS84; **Identification:** identifiedBy: A. Polevoi; **Event:** samplingProtocol: Trunk emergence trap; eventDate: 2016-07-21/08-1; **Record Level:** institutionCode: FRIP
- **Type status:** Other material. **Occurrence:** recordedBy: A. Polevoi; sex: 95 males, 86 females; **Location:** country: Russia; stateProvince: Karelia; locality: Kivach Nature Reserve; verbatimLatitude: 62.281; verbatimLongitude: 33.967; verbatimCoordinateSystem: Decimal degrees; verbatimSRS: WGS84; **Identification:** identifiedBy: A. Polevoi; **Event:** samplingProtocol: Trunk emergence trap; eventDate: 2016-08-19/09-28; **Record Level:** institutionCode: FRIP

#### Diagnosis

**Figure 4.**
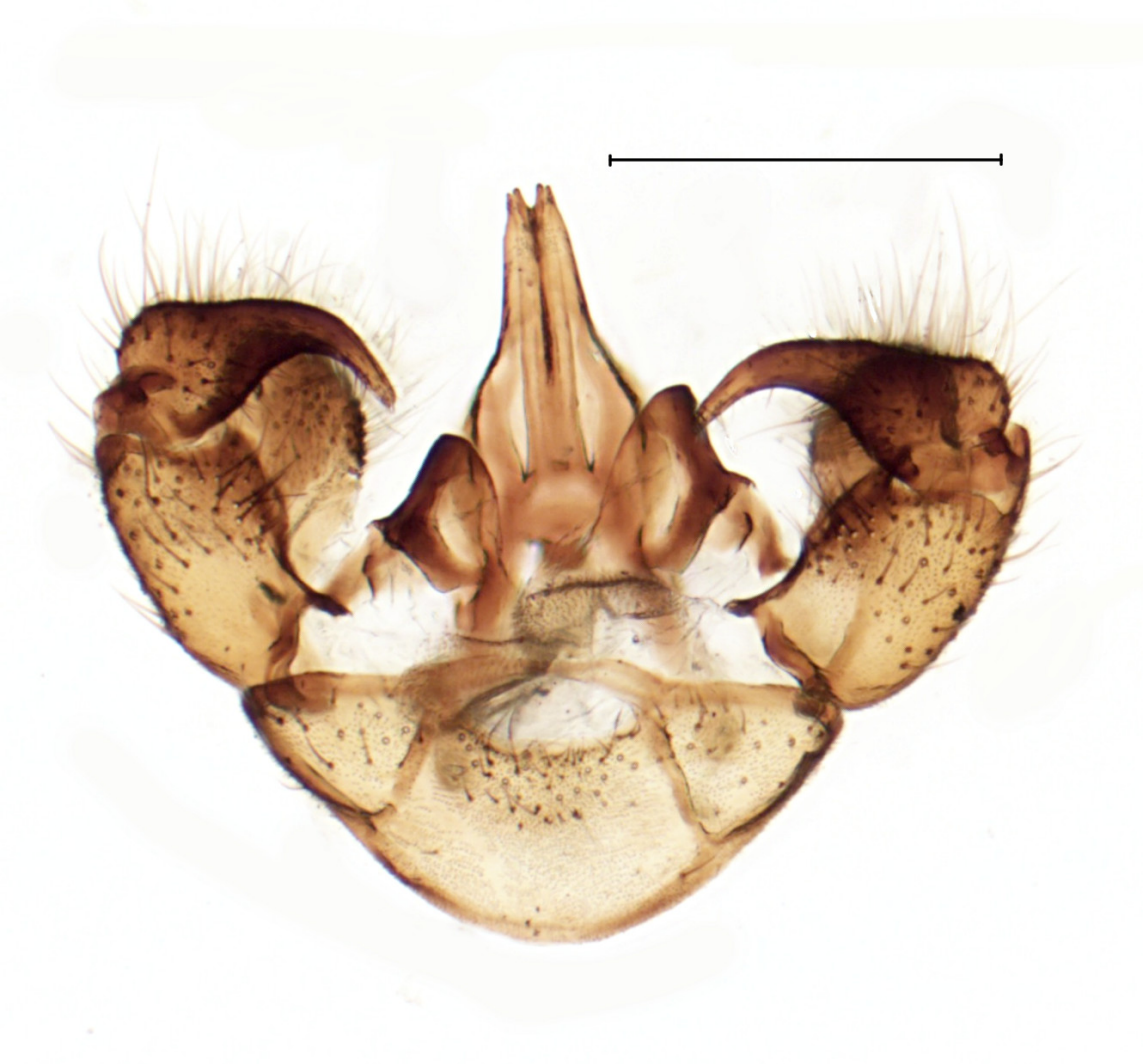
*Limonia badia* (Walker), male genitalia, dorsal view. Scale bar: 0.5 mm.

Distinguished from other *Limonia* by the structure of the male genitalia (Fig. 4) as well as wing pattern and the shape of the tarsal claws (Starý and Salmela 2004, fig. 1, 2).

#### Distribution

Holarctic species, recently discovered in Finland (Starý and Salmela 2004) and Russian Karelia (Polevoi and Salmela 2014).

#### Ecology

Saproxylic species, associated with dead aspen wood (Halme et al. 2012). According to the authors' data, this species prefers wind-broken aspens with a diameter over 50 cm, fallen 6 years ago Table 1. It favoured the trunks colonised by *Hyphoderma setigerum* (RS=0.81, P<0.01), *Peniophora incarnata* (RS=0.62, P<0.05), *Scutellinia scutellata* and *Lentaria afflata* (RS=0.59, P<0.05).

#### Conservation

Red-listed in Finland, category NT (Penttinen et al. 2010).

### Ectaetia subclavipes

Krivosheina, 2002

http://www.catalogueoflife.org/annual-checklist/2017/details/species/id/4d727f0ceba0ba5043f0f48b85d53d30

#### Materials

- **Type status:** Other material. **Occurrence:** recordedBy: A. Polevoi; sex: 98 males, 135 females; **Location:** country: Russia; stateProvince: Karelia; locality: Kivach Nature Reserve; verbatimLatitude: 62.281; verbatimLongitude: 33.967; verbatimCoordinateSystem: Decimal degrees; verbatimSRS: WGS84; **Identification:** identifiedBy: A. Polevoi; **Event:** samplingProtocol: Trunk emergence trap; eventDate: 2016-05-26/06-23; **Record Level:** institutionCode: FRIP
- **Type status:** Other material. **Occurrence:** recordedBy: A. Polevoi; sex: 11 males, 95 females; **Location:** country: Russia; stateProvince: Karelia; locality: Kivach Nature Reserve; verbatimLatitude: 62.281; verbatimLongitude: 33.967; verbatimCoordinateSystem: Decimal degrees; verbatimSRS: WGS84; **Identification:** identifiedBy: A. Polevoi; **Event:** samplingProtocol: Trunk emergence trap; eventDate: 2016-06-23/07-21; **Record Level:** institutionCode: FRIP
- **Type status:** Other material. **Occurrence:** recordedBy: A. Polevoi; sex: 1 male, 1 female; **Location:** country: Russia; stateProvince: Karelia; locality: Kivach Nature Reserve; verbatimLatitude: 62.281; verbatimLongitude: 33.967; verbatimCoordinateSystem: Decimal degrees; verbatimSRS: WGS84; **Identification:** identifiedBy: A. Polevoi; **Event:** samplingProtocol: Trunk emergence trap; eventDate: 2016-07-21/08-19; **Record Level:** institutionCode: FRIP

#### Diagnosis

**Figure 5.**
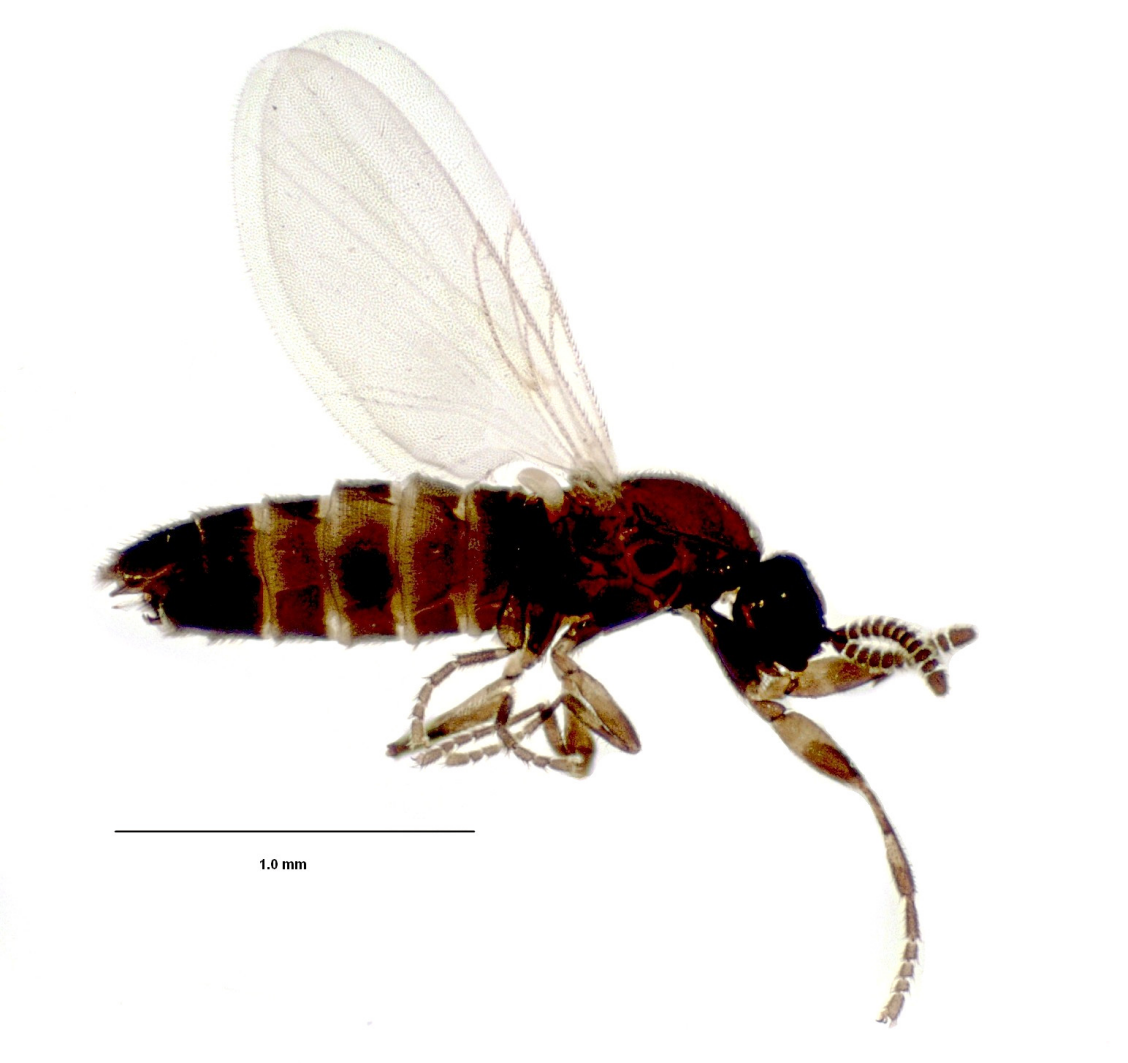
*Ectaetia subclavipes* Krivosheina, habitus.

Flies of the genus *Ectaetia* have peculiar wing venation, namely the vein M partly fused with R_4+5_ (Fig. 5). *Ectaetia subclavipes* is distinguished from congeners by the structure of the male genitalia (Fig. 6; see also Krivosheina (2002), fig. 28-30).

#### Distribution

East Russia: Khabarovsk region, Amur province, Tuva, Sakhalin island (Krivosheina 2002). First record for Europe.

#### Ecology

Larvae develop under the bark of aspen and poplar (Krivosheina 2002). According to the authors' data, the species prefers huge wind-broken aspens (diameter 70 cm. and more) fallen 7-10 years ago Table 1. It favoured the trunks colonised by *Oxyporus corticola* (RS=0.69, P<0.01), *Trametes ochracea* (RS=0.63, P<0.05) and *Hyphoderma setigerum* (RS=0.56, P<0.05).

### Mesaxymyia kerteszi

(Duda, 1930)

http://www.catalogueoflife.org/annual-checklist/2017/details/species/id/8e5f999d7fd9b092a5b50a3bdf7fef17

#### Materials

- **Type status:** Other material. **Occurrence:** recordedBy: A. Polevoi; sex: 1 female; **Location:** country: Russia; stateProvince: Karelia; locality: Kivach Nature Reserve; verbatimLatitude: 62.281; verbatimLongitude: 33.967; verbatimCoordinateSystem: Decimal degrees; verbatimSRS: WGS84; **Identification:** identifiedBy: A. Polevoi; **Event:** samplingProtocol: Trunk emergence trap; eventDate: 2016-05-26/06-2; **Record Level:** institutionCode: FRIP

#### Diagnosis

**Figure 7.**
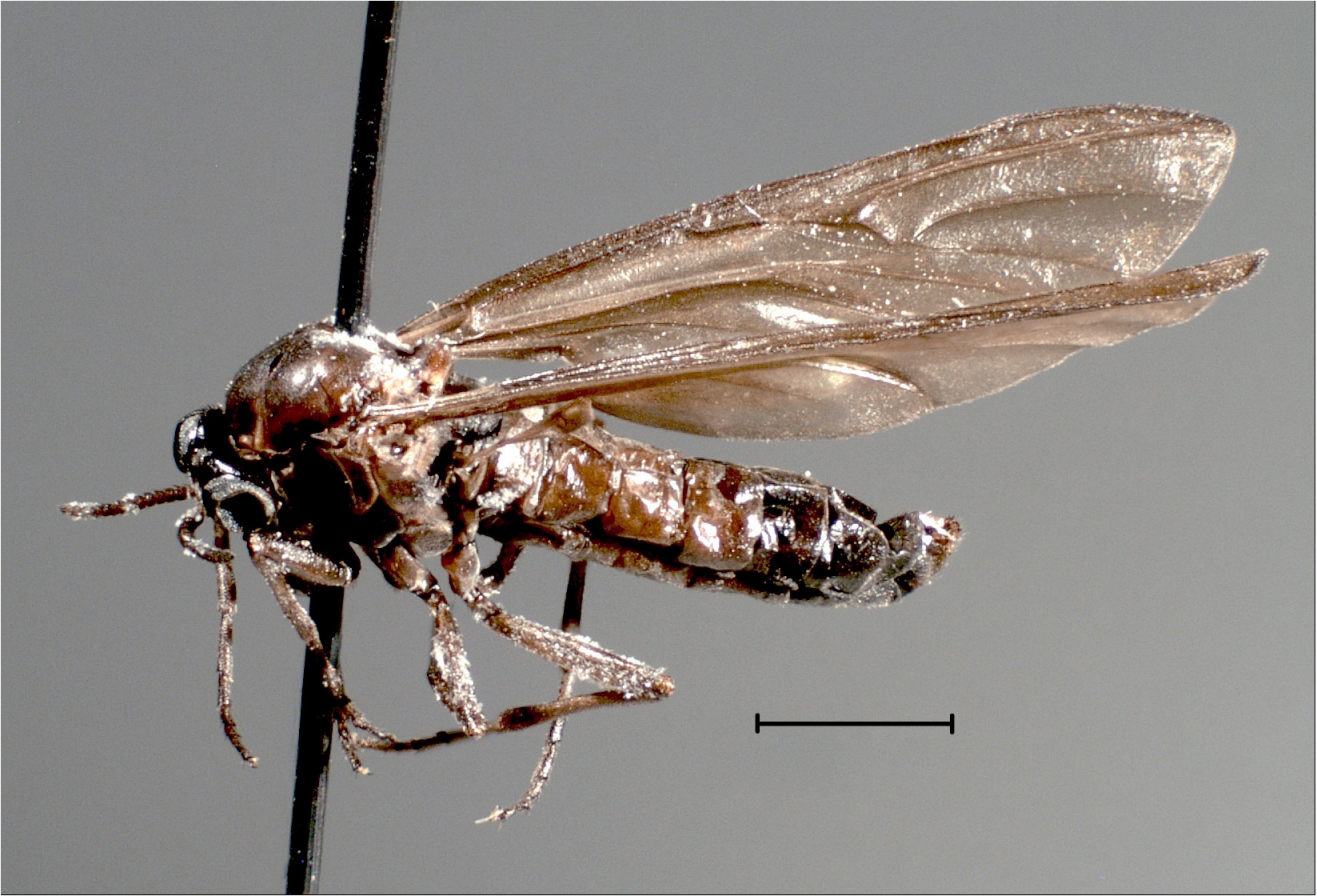
*Mesaxymyia kerteszi* (Duda), female habitus. Scale bar: 2 mm.

Medium-sized flies with darkened wings (Fig. 7).

#### Distribution

Europe. Known by a few records from the European part of Russia, Ukraine and Slovakia (Humala and Polevoi 2009, Jakovlev and Polevoi 1997, Martinovský and Roháček 1993).

#### Ecology

Poorly known. Larvae were collected from spruce wood (Mamaev and Krivosheina 1966) and adults found sitting on very damp fir logs (Martinovský and Roháček 1993). According to Krivosheina (1991), larvae of Axymyiidae may colonise newly fallen trunks and stay there for several years. This species was collected from a wind-broken aspen, colonised by 7 species of macrofungi Table 1.

### Impleta consorta

Plassmann, 1978

http://www.catalogueoflife.org/annual-checklist/2017/details/species/id/0e3b60c045c2ad149fb1c41f3bdb2b06

#### Materials

- **Type status:** Other material. **Occurrence:** recordedBy: A. Polevoi; sex: 1 male,; **Location:** country: Russia; stateProvince: Karelia; locality: Kivach Nature Reserve; verbatimLatitude: 62.281; verbatimLongitude: 33.967; verbatimCoordinateSystem: Decimal degrees; verbatimSRS: WGS84; **Identification:** identifiedBy: A. Polevoi; **Event:** samplingProtocol: Trunk emergence trap; eventDate: 2016-07-21/08-1; **Record Level:** institutionCode: FRIP

#### Diagnosis

Small gnat, distinguished by wing venation (see http://sciaroidea.info/taxonomy/42011) and characters of the male genitalia (Fig. 8; see also Matile (1983), fig. 1).

#### Distribution

Palaearctic. Known by a few specimens from Sweden, Poland, Finland and Norway (Kurina 2003, J. Kjærandsen pers. comm.). Recently reported from East Russia (Polevoi and Barkalov 2017).

#### Ecology

Poorly known. Some specimens were caught with light traps (Plassmann 1980, Kurina 2003, J. Kjærandsen pers. comm.). This species was collected from a wind-broken aspen, colonised by 15 species of macrofungi Table 1.

#### Conservation

Red-listed in Finland, category NT (Penttinen et al. 2010).

### Zygoneura (Pharetratula) bidens

(Mamaev, 1968)

http://www.catalogueoflife.org/annual-checklist/2017/details/species/id/237e139b55f7d5dd99863fe5e71a4491

#### Materials

- **Type status:** Other material. **Occurrence:** recordedBy: A. Polevoi; sex: 1 male; **Location:** country: Russia; stateProvince: Karelia; locality: Kivach Nature Reserve; verbatimLatitude: 62.281; verbatimLongitude: 33.967; verbatimCoordinateSystem: Decimal degrees; verbatimSRS: WGS84; **Identification:** identifiedBy: A. Polevoi; **Event:** samplingProtocol: Trunk emergence trap; eventDate: 2016-05-26/06-23; **Record Level:** institutionCode: FRIP
- **Type status:** Other material. **Occurrence:** recordedBy: A. Polevoi; sex: 1 male; **Location:** country: Russia; stateProvince: Karelia; locality: Kivach Nature Reserve; verbatimLatitude: 62.281; verbatimLongitude: 33.967; verbatimCoordinateSystem: Decimal degrees; verbatimSRS: WGS84; **Identification:** identifiedBy: A. Polevoi; **Event:** samplingProtocol: Trunk emergence trap; eventDate: 2016-06-23/07-21; **Record Level:** institutionCode: FRIP
- **Type status:** Other material. **Occurrence:** recordedBy: A. Polevoi; sex: 13 males; **Location:** country: Russia; stateProvince: Karelia; locality: Kivach Nature Reserve; verbatimLatitude: 62.281; verbatimLongitude: 33.967; verbatimCoordinateSystem: Decimal degrees; verbatimSRS: WGS84; **Identification:** identifiedBy: A. Polevoi; **Event:** samplingProtocol: Trunk emergence trap; eventDate: 2016-07-21/08-19; **Record Level:** institutionCode: FRIP
- **Type status:** Other material. **Occurrence:** recordedBy: A. Polevoi; sex: 1 male; **Location:** country: Russia; stateProvince: Karelia; locality: Kivach Nature Reserve; verbatimLatitude: 62.281; verbatimLongitude: 33.967; verbatimCoordinateSystem: Decimal degrees; verbatimSRS: WGS84; **Identification:** identifiedBy: A. Polevoi; **Event:** samplingProtocol: Trunk emergence trap; eventDate: 2016-08-19/09-28; **Record Level:** institutionCode: FRIP

#### Diagnosis

Gnats of the genus *Zygoneura* are distinguished from other Sciaridae by widely curved fork of wing vein M and long neck of ﬂagellomeres (Fig. 9a). *Zygoneura bidens* can be recognised by the two dark spines on a shared lobe in the middle of gonostylus (Fig. 9b; see also Shin et al. (2014), fig. 1: A, B).

#### Distribution

Palaearctic: Slovakia (Rudzinski and Ševčík 2012), China, Korea and the Russian Far East (Shin et al. 2014).

#### Ecology

Evidently saproxylic as other species of the subgenus Pharetratula. In Korea, collected at a shiitake farm with oak trunks used as a substrate for shiitake culture (Shin et al. 2014). According to the authors' data, the species prefers wind-broken aspens with a mean diameter of ca. 40 cm and having died 9 years ago Table 1. It favoured the trunks colonised by *Peniophora incarnata* (RS=0.61, P<0.05).

### Agathomyia vernalis

Shatalkin, 1981

http://www.catalogueoflife.org/annual-checklist/2017/details/species/id/749f7ac63c3cea4cc9f79d6f4d701a96

#### Materials

- **Type status:** Other material. **Occurrence:** recordedBy: A. Polevoi; sex: 2 males, 8 females; **Location:** country: Russia; stateProvince: Karelia; locality: Kivach Nature Reserve; verbatimLatitude: 62.281; verbatimLongitude: 33.967; verbatimCoordinateSystem: Decimal degrees; verbatimSRS: WGS84; **Identification:** identifiedBy: A. Polevoi; **Event:** samplingProtocol: Trunk emergence trap; eventDate: 2016-04-25/05-26; **Record Level:** institutionCode: FRIP

#### Diagnosis

**Figure 10.**
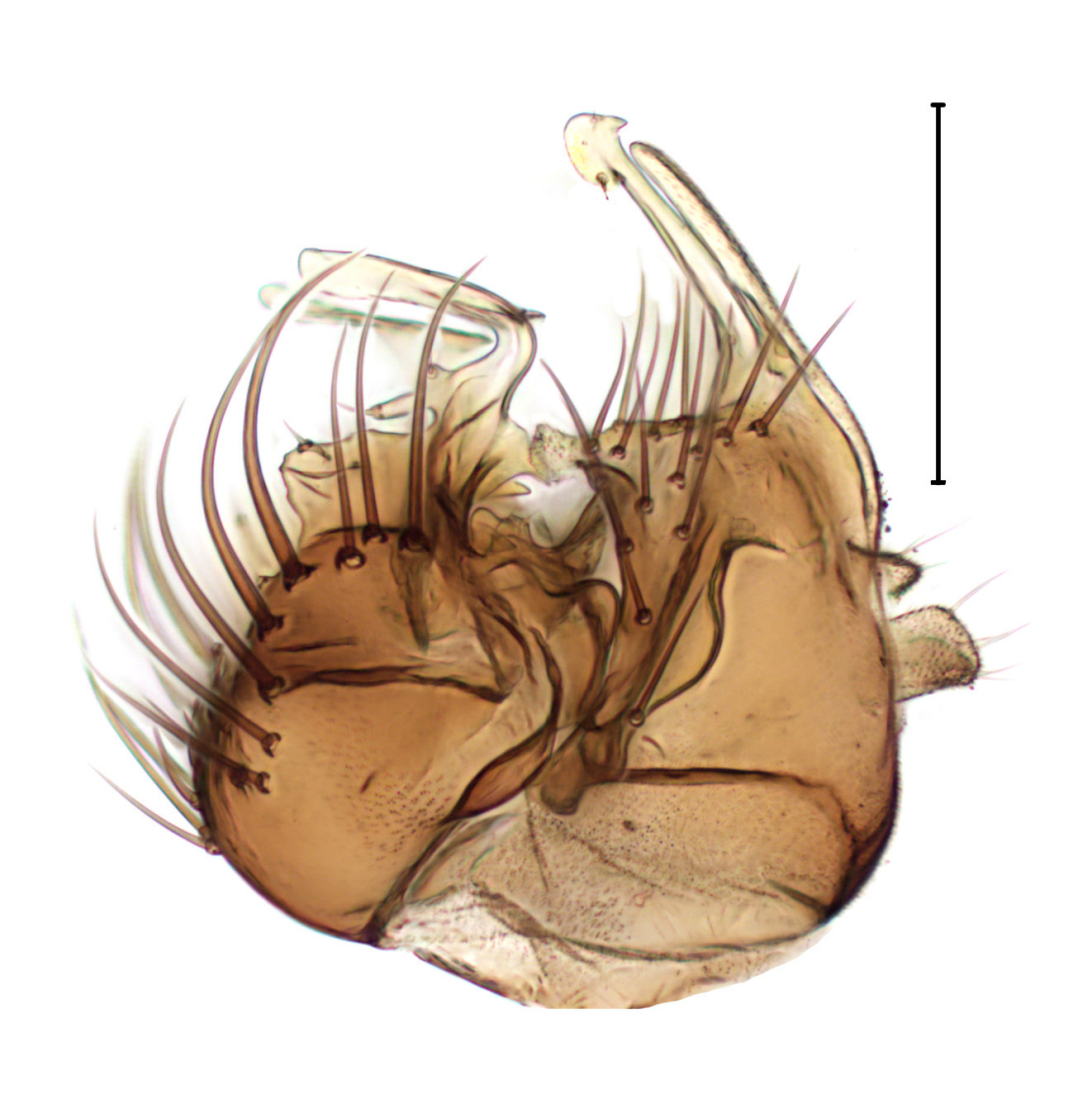
*Agathomyia vernalis* Shatalkin, male genitalia, lateral view. Scale bar: 0.2 mm.

Males of *A. vernalis* are distinguished from congeners by several morphological characters, including the structure of the male genitalia (Fig. 10; see also Chandler (2001), Fig. 107-109), while females are recognised by peculiar abdominal marking (Chandler 2001, fig. 110, 111).

#### Distribution

Europe: Moscow province of Russia, Czech and Slovak Republics, Switzerland (Chandler 2001), Finland (Ståhls and Kahanpää 2006) and Romania (Tkoč and Roháček 2014).

#### Ecology

Virtually unknown. Other *Agathomyia* species assumed to develop internally in polypore fungi (Chandler 2001). In the Czech Republic, adults were registered in pyramidal (emergence) traps (Tkoč and Barták 2013). This species was collected from aspens fallen 8-10 years ago. The species did not show any preferences either to the trunk size or tree mortality mode (windfall vs. windbreak) Table 1. Its occurrence positively correlated with the following fungi: *Fomitopsis pinicola* (RS=0.99, P<0.01), *Cerioporus leptocephalus*, *Royoporus badius*, *Trametes trogii*, *Punctularia strigosozonata* (RS=0.73, P<0.01), *Xenasma rimicola*, *Kurtia argillacea*, *Resinicium bicolor*, *Tomentella badia*, *T. crinalis* and *T. lapida* (RS=0.61, P<0.05).

#### Conservation

Red-listed in Czech Republic, category EN (Farkac et al. 2005).

### Hammerschmidtia ingrica

Stackelberg, 1952

https://fauna-eu.org/cdm_dataportal/taxon/197de867-9c6b-4f01-9aa3-410813b7d47f

#### Materials

- **Type status:** Other material. **Occurrence:** recordedBy: A. Polevoi; sex: 3 males, 1 females; **Location:** country: Russia; stateProvince: Karelia; locality: Kivach Nature Reserve; verbatimLatitude: 62.281; verbatimLongitude: 33.967; verbatimCoordinateSystem: Decimal degrees; verbatimSRS: WGS84; **Identification:** identifiedBy: A. Polevoi; **Event:** samplingProtocol: Trunk emergence trap; eventDate: 2016-04-25/05-26; **Record Level:** institutionCode: FRIP

#### Diagnosis

Easily distinguished from more common *H. ferruginea* Fallén by smaller body size and short feathering of the arista (Fig. 11, see also Kerppola (2011), fig. 2-4).

#### Distribution

Palaearctic. Few specimens are known from Finland and Leningrad province of Russia (Kerppola 2011). In East Russia, recorded from the republic of Tyva to Primorje region (Krivosheina 2003).

#### Ecology

Saproxylic species, associated with deciduous trees (Krivosheina 2003). This fly was collected from a wind-broken aspen, colonised by 7 species of macrofungi Table 1.

#### Conservation

Red-listed in Leningrad province of Russia (Krivokhatsky and Kuznetsov 2002).

### Clusiodes tuomikoskii

Mamaev, 1974

http://www.catalogueoflife.org/annual-checklist/2017/details/species/id/56f936b25a743fb51421c5ddb9a880a0

#### Materials

- **Type status:** Other material. **Occurrence:** recordedBy: A. Polevoi; sex: 14 males, 14 females; **Location:** country: Russia; stateProvince: Karelia; locality: Kivach Nature Reserve; verbatimLatitude: 62.281; verbatimLongitude: 33.967; verbatimCoordinateSystem: Decimal degrees; verbatimSRS: WGS84; **Identification:** identifiedBy: A. Polevoi; **Event:** samplingProtocol: Trunk emergence trap; eventDate: 2016-04-25/05-26; **Record Level:** institutionCode: FRIP

#### Diagnosis

**Figure 12.**
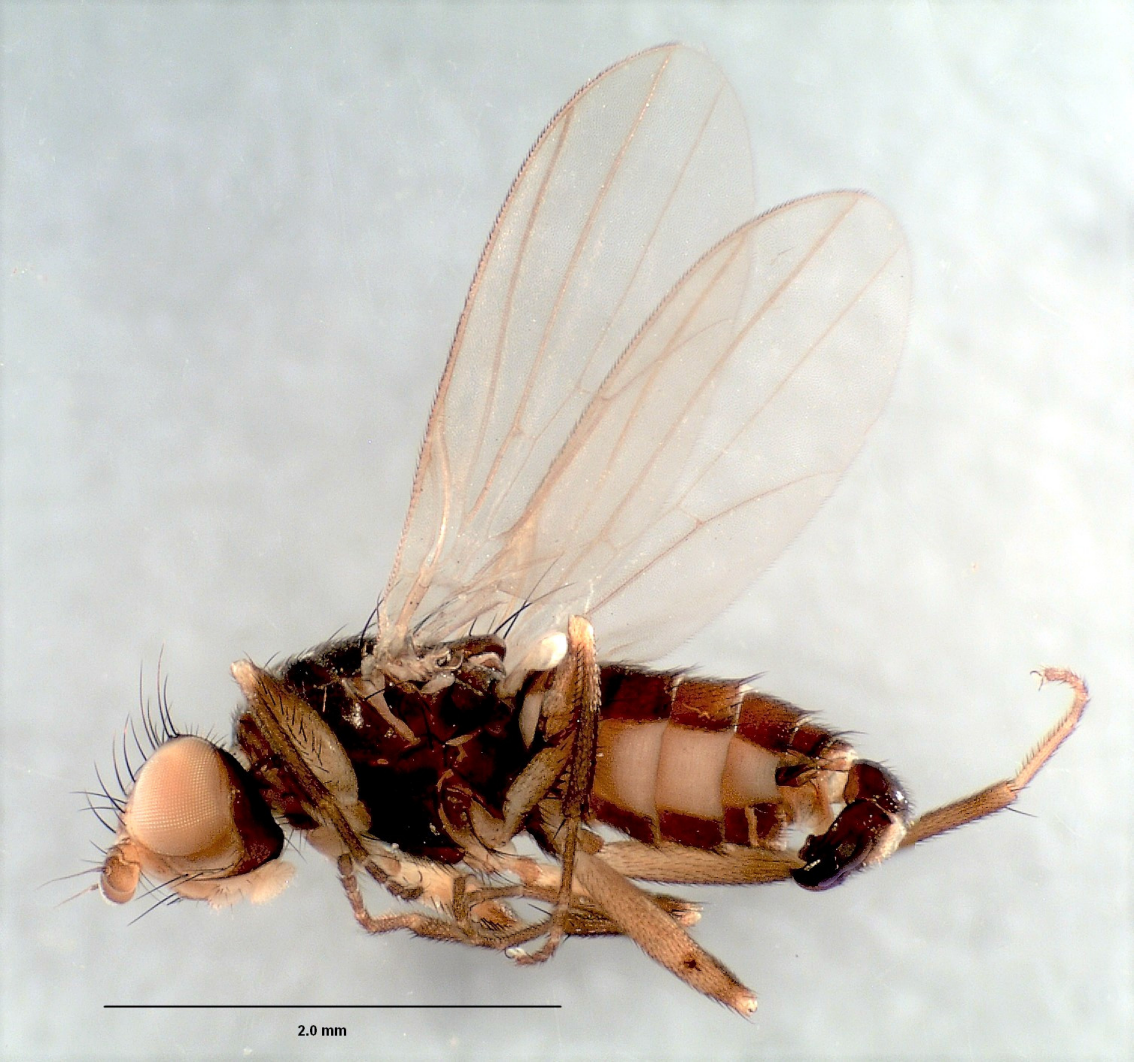
*Clusiodes tuomikoskii* Mamaev, male habitus.

The only representative of the genus *Clusiodes* with no darkening along the costal vein and entirely black scutum (Fig. 12). Male surstylus figured by Mamaev (1974), fig. 1:3 and Lonsdale and Marshall (2007), fig. 41.

#### Distribution

Known only by the type specimens from East Russia, Primorje region (Lonsdale and Marshall 2007, Mamaev 1974). First record for Europe.

#### Ecology

Unknown, but most probably similar to other species of *Clusiodes*, which develop in dead wood in an advanced state of decay, preferring fallen bark-covered trunks or stumps (Rotheray and Horsfield 2013). According to the authors' data, this species prefers wind-broken aspens of average diameter (ca. 40 cm) fallen 9 years ago (Table 1). It favoured the trunks colonised by *Peniophora incarnata* (RS=0.61, P<0.05).

## Discussion

Trunk emergence traps have some benefits in comparison with other methods traditionally used for collection of wood-living Coleoptera and Diptera (Alinvi et al. 2006, Halme et al. 2012). They, however fail in some cases (e.g. when larvae need to migrate to the soil for pupation) and usually cannot provide exact information about microhabitat preferences (Økland 1999). These traps were shown to be effective in detecting rather diverse insect communities, including a range of rare and otherwise remarkable species, which are difficult to find using commonly used techniques (Polevoi et al. 2017). This study once more demonstrates the effectiveness of the method and also shows the importance of large dead aspens for supporting diverse saproxylic insect communities. It is realised that indirect association of Diptera species with certain trunk parameters or fungi, e.g. correlation, may be questionable because of the relatively small number of replicates. However, in case of species with poorly known biology, such information may be of great importance. Initial knowledge about species preferences may help to outline directions for future studies and conservation measures.

## Supplementary Material

Supplementary material 1 The list of Diptera species collected with trunk emergence traps Data type: occurrences
Brief description: The list of species and number of collected specimens.
File: oo_178484.xls Alexei Polevoi

XML Treatment for Gnophomyia acheron

XML Treatment for Atypophthalmus (Microlimonia) machidai

XML Treatment for Discobola parvispinula

XML Treatment for Limonia badia

XML Treatment for Ectaetia subclavipes

XML Treatment for Mesaxymyia kerteszi

XML Treatment for Impleta consorta

XML Treatment for Zygoneura (Pharetratula) bidens

XML Treatment for Agathomyia vernalis

XML Treatment for Hammerschmidtia ingrica

XML Treatment for Clusiodes tuomikoskii

## Figures and Tables

**Figure 1a.**
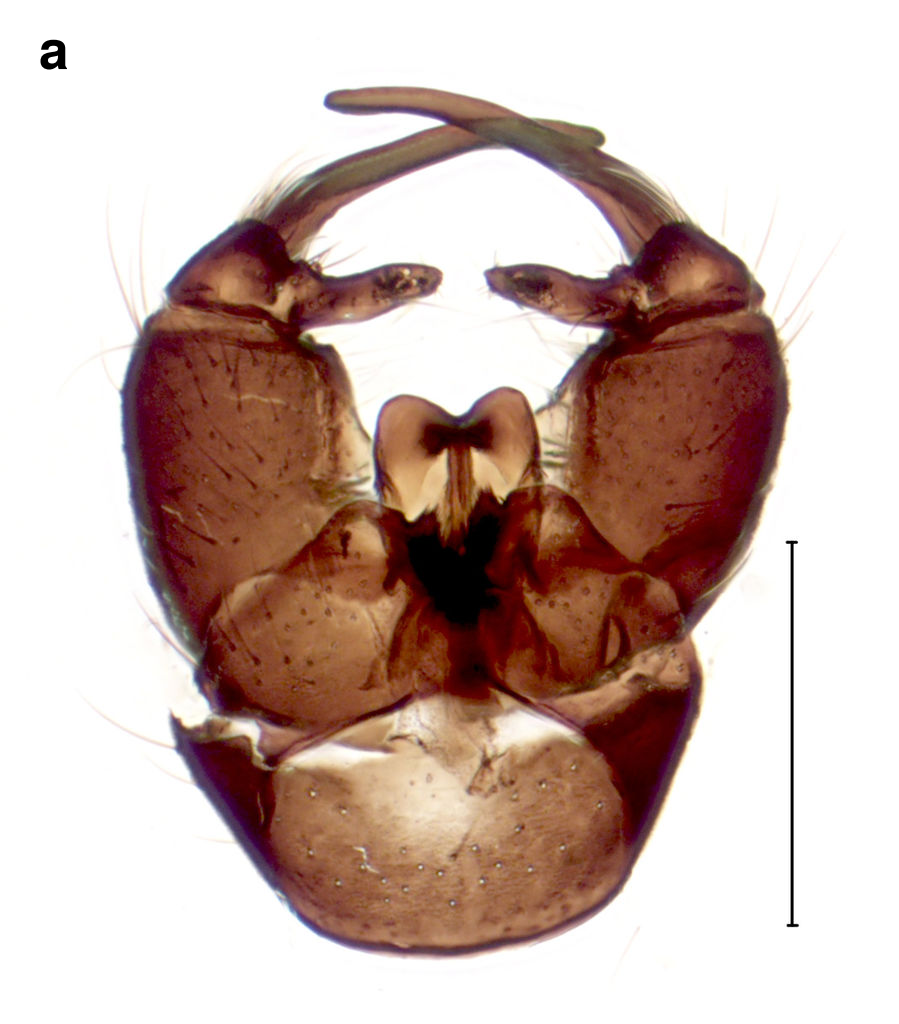
Dorsal view

**Figure 1b.**
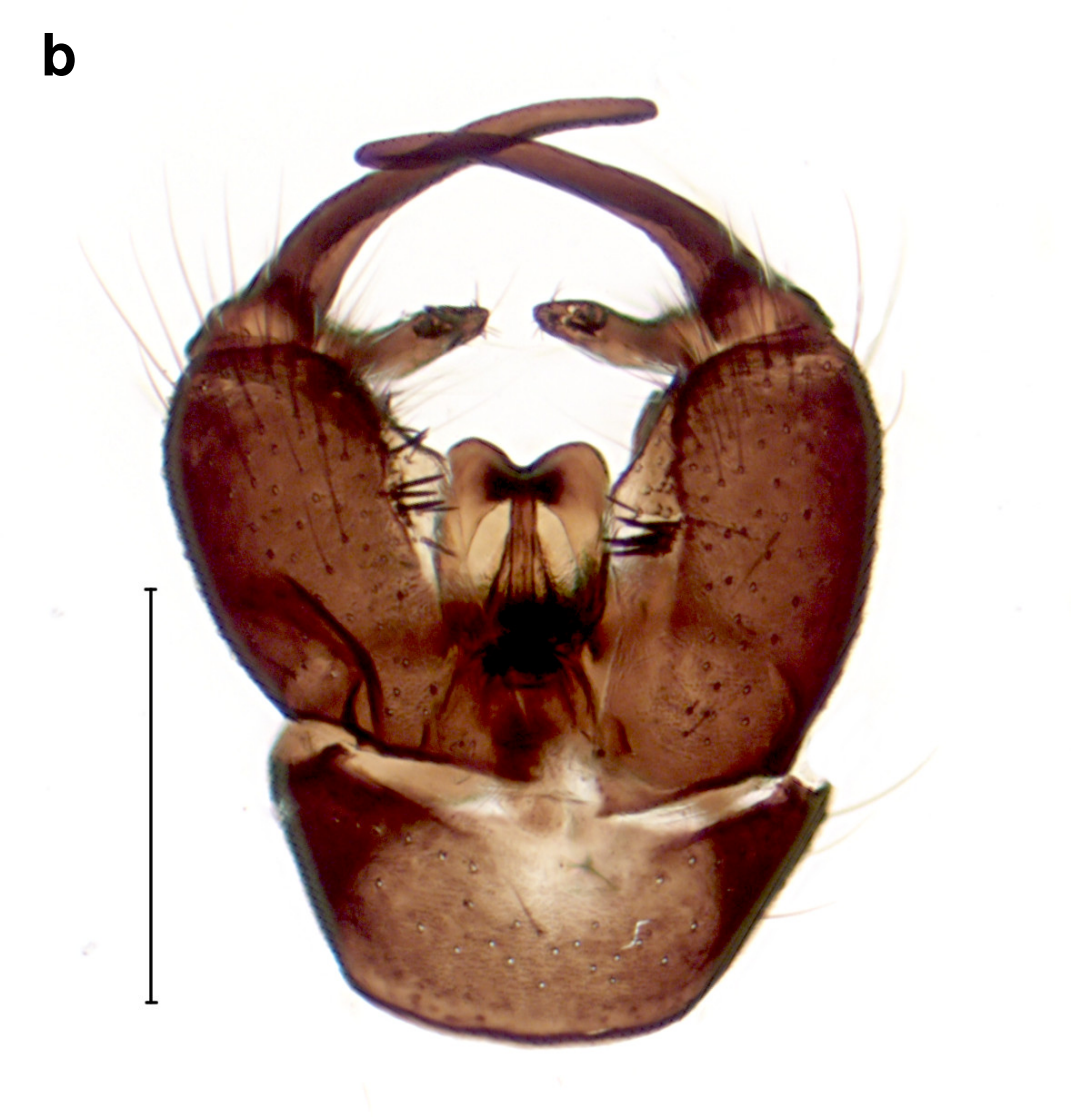
Ventral view

**Figure 2a.**
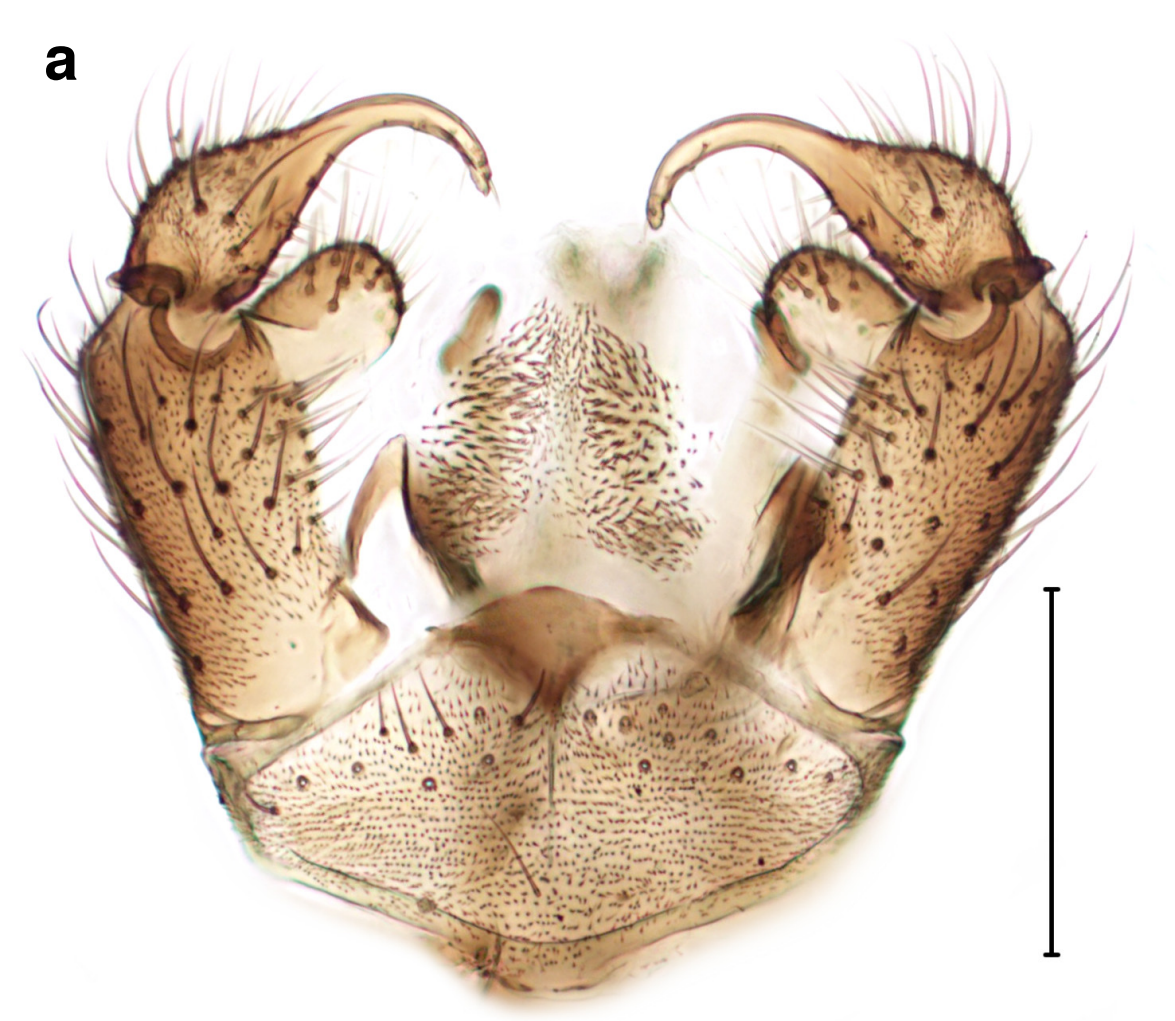
Dorsal view.

**Figure 2b.**
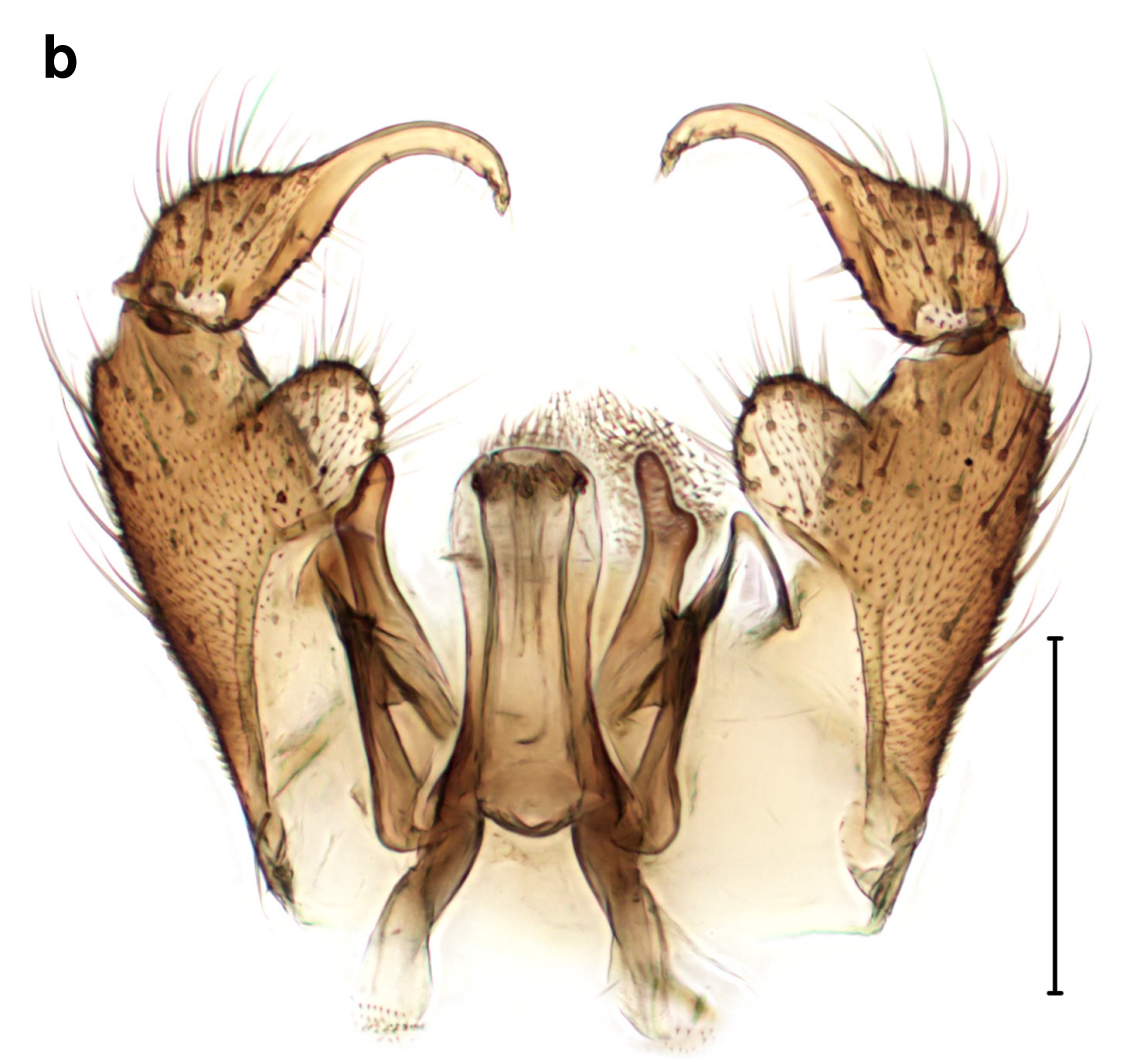
Ventral view.

**Figure 3a.**
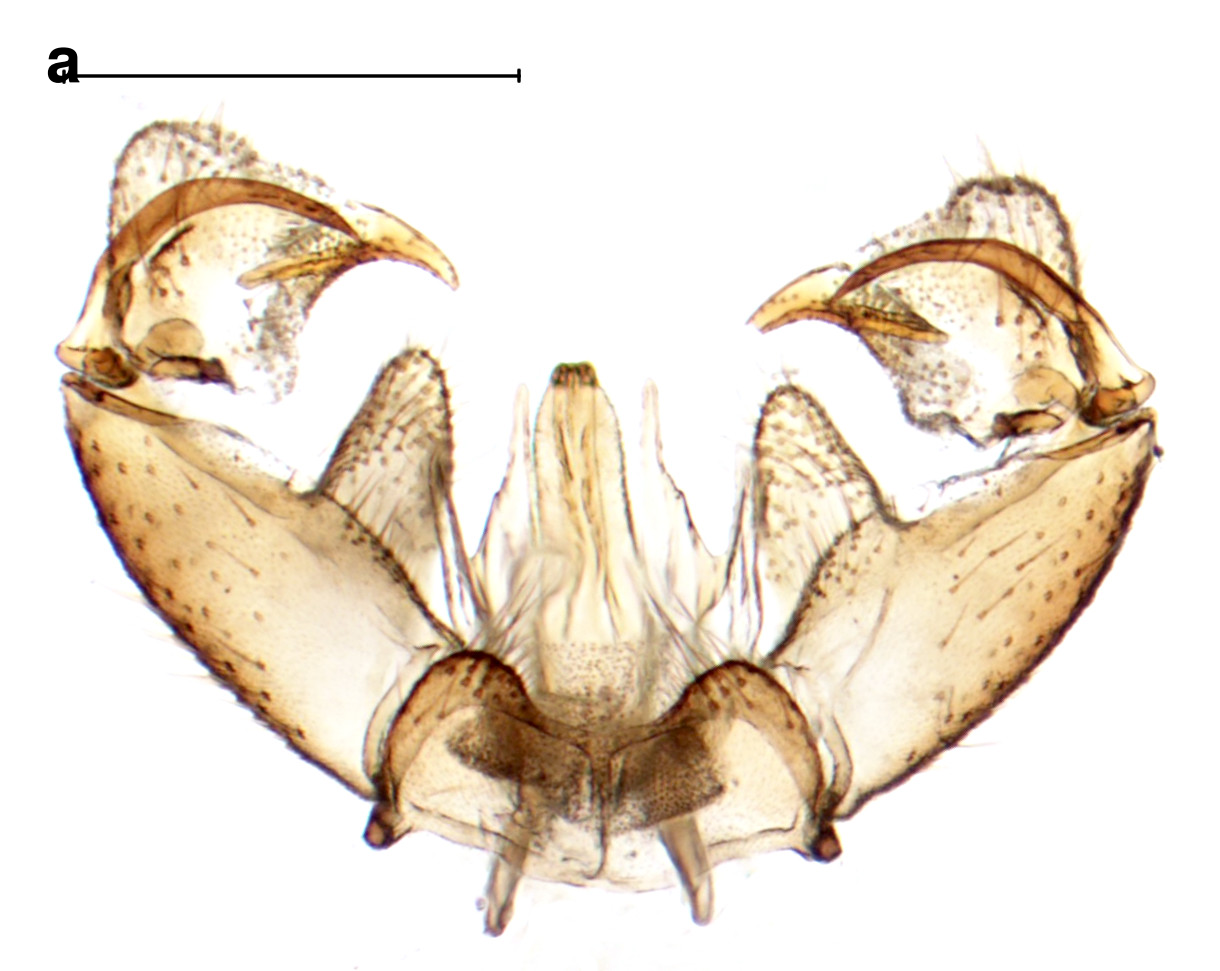
Dorsal view. Scale bar: 0.5 mm.

**Figure 3b.**
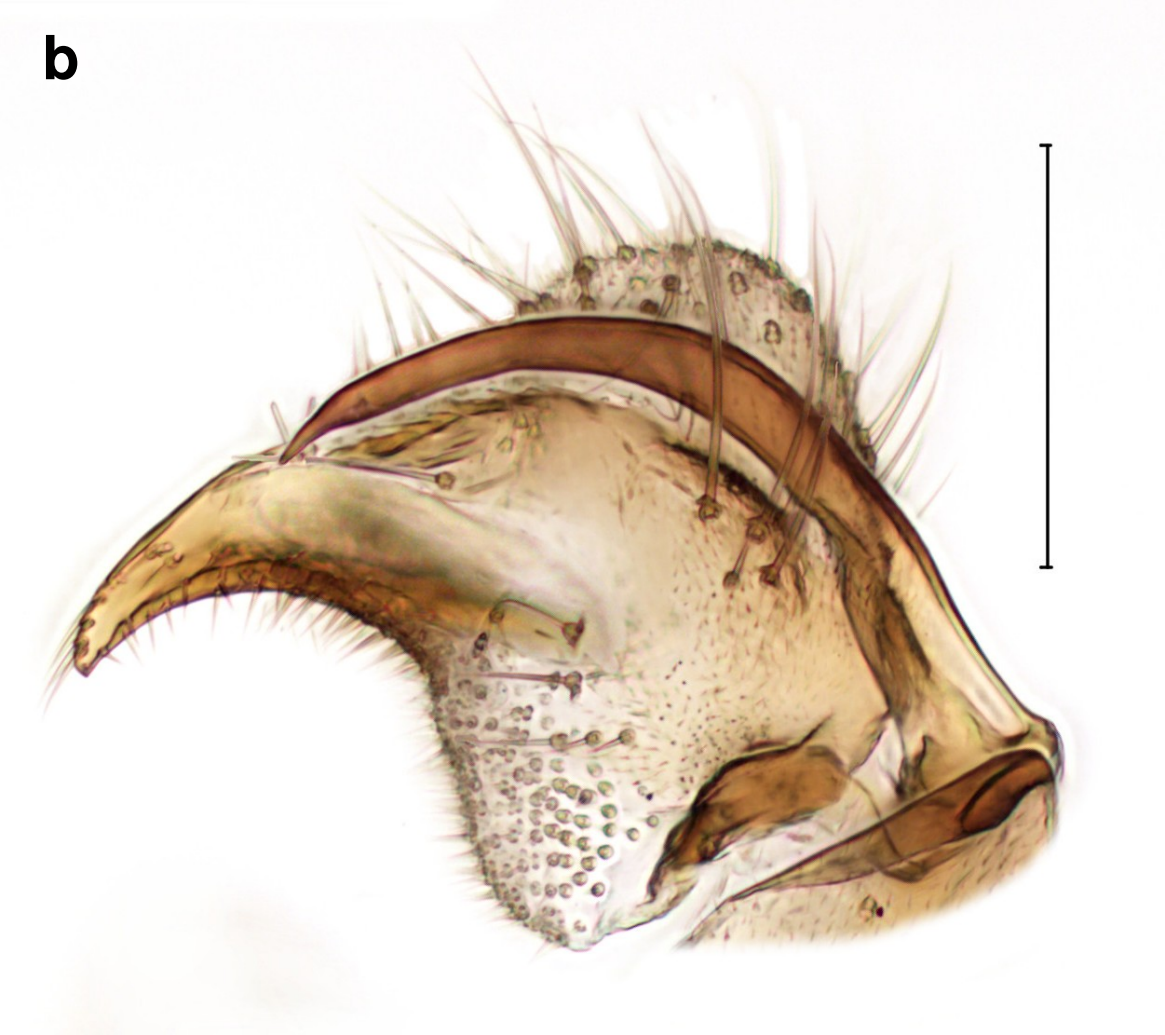
Gonostylus. Scale bar: 0.2 mm.

**Figure 6a.**
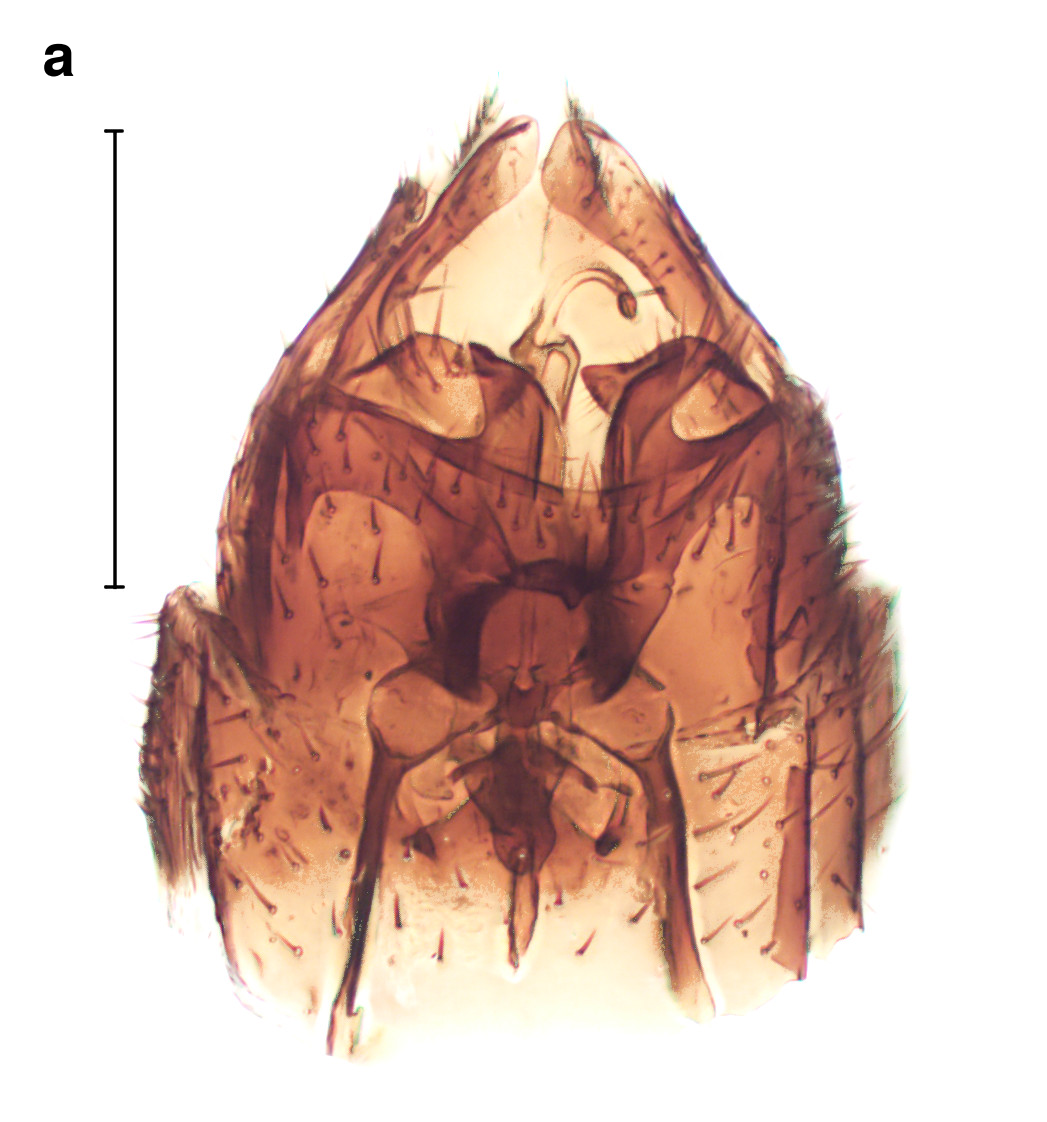
Ventral view.

**Figure 6b.**
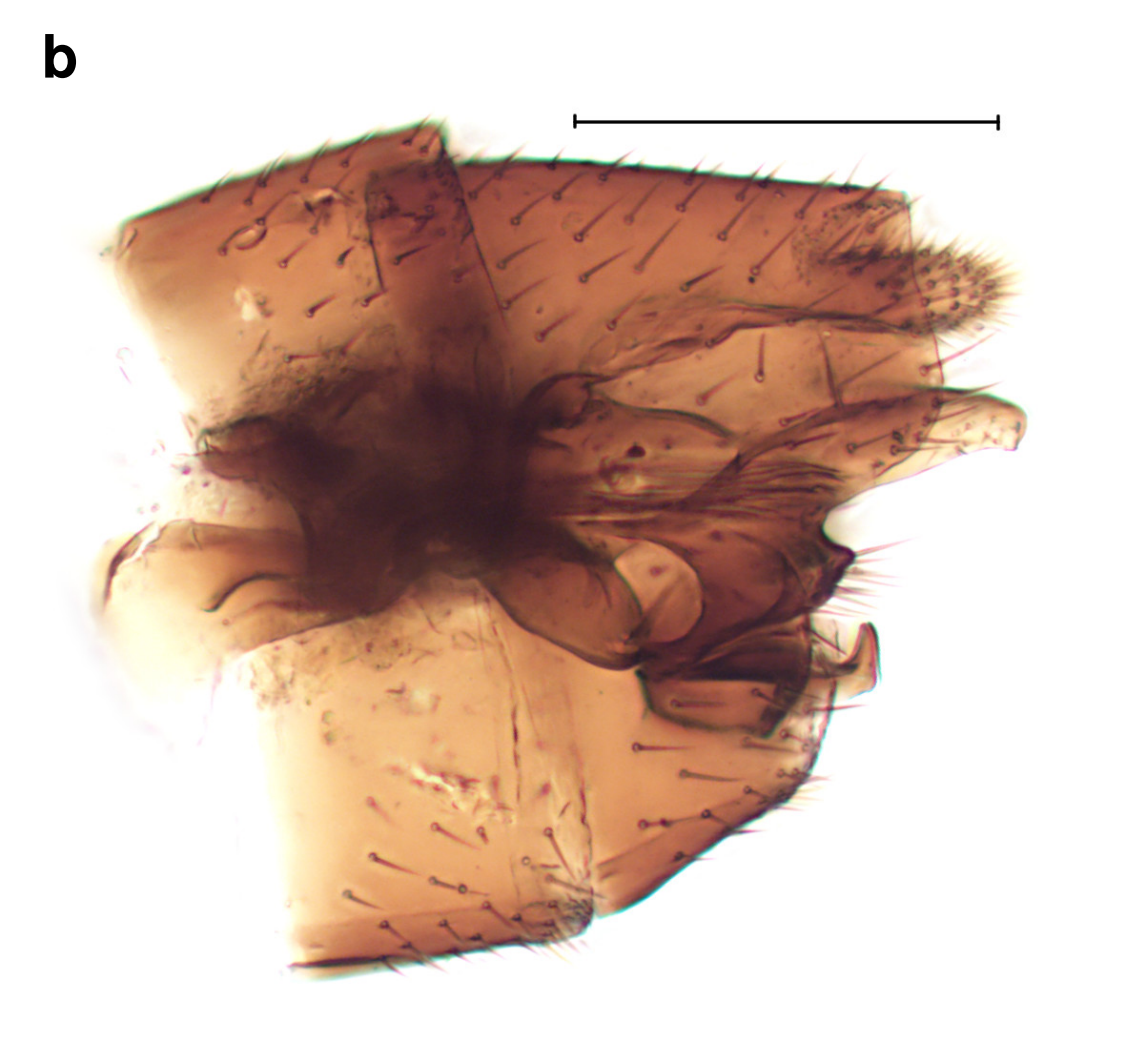
Lateral view.

**Figure 8a.**
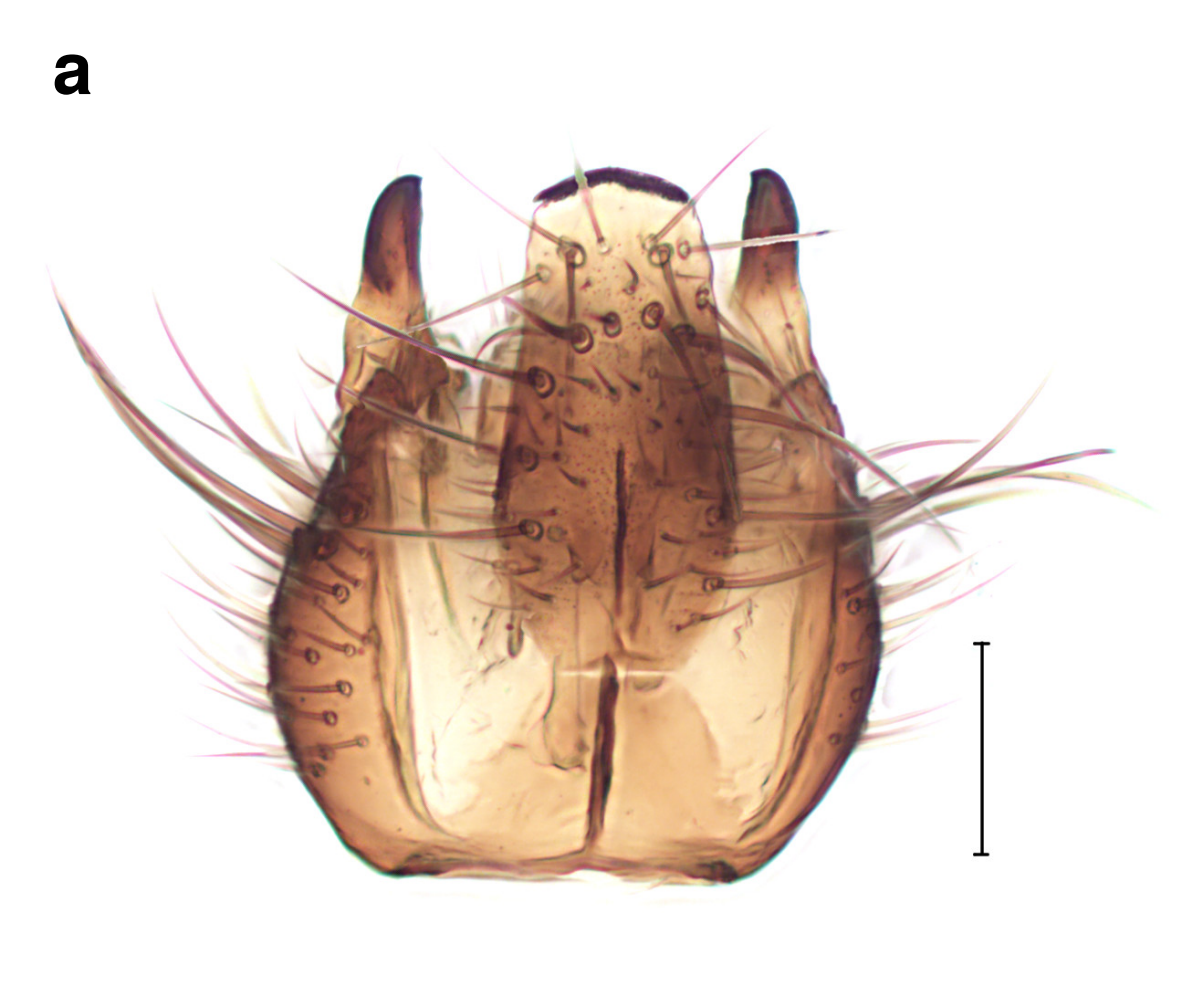
Dorsal view.

**Figure 8b.**
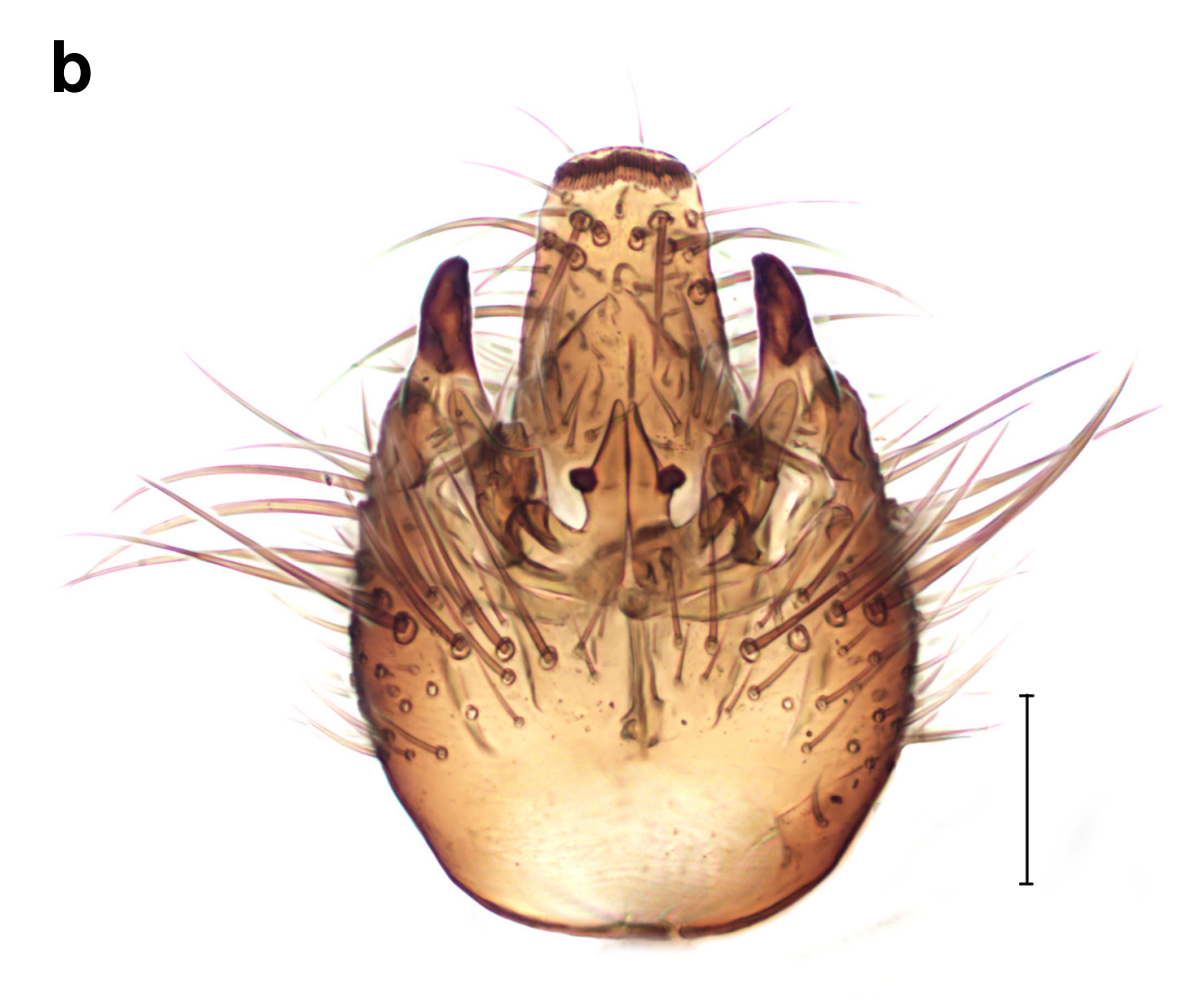
Ventral view.

**Figure 9a.**
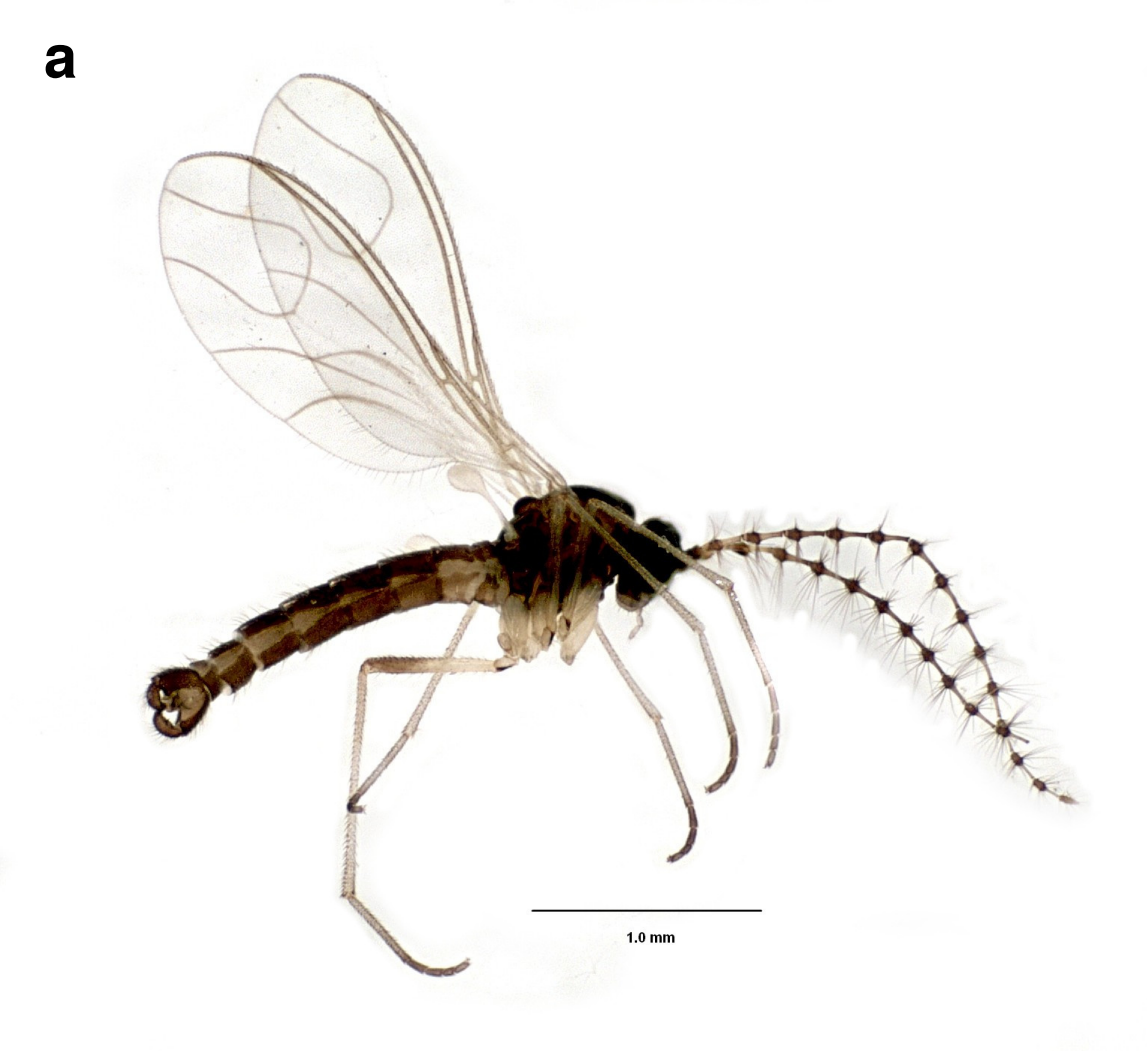
Habitus.

**Figure 9b.**
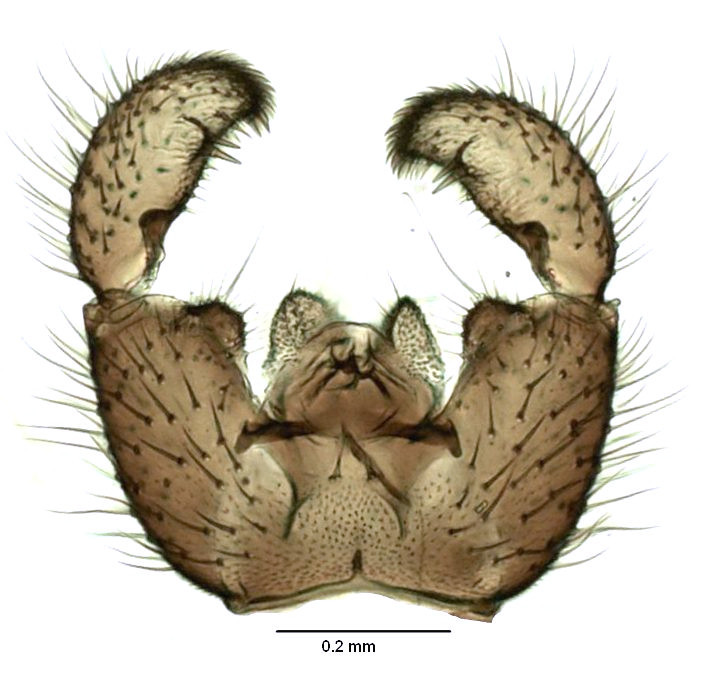
Genitalia, ventral view.

**Figure 11a.**
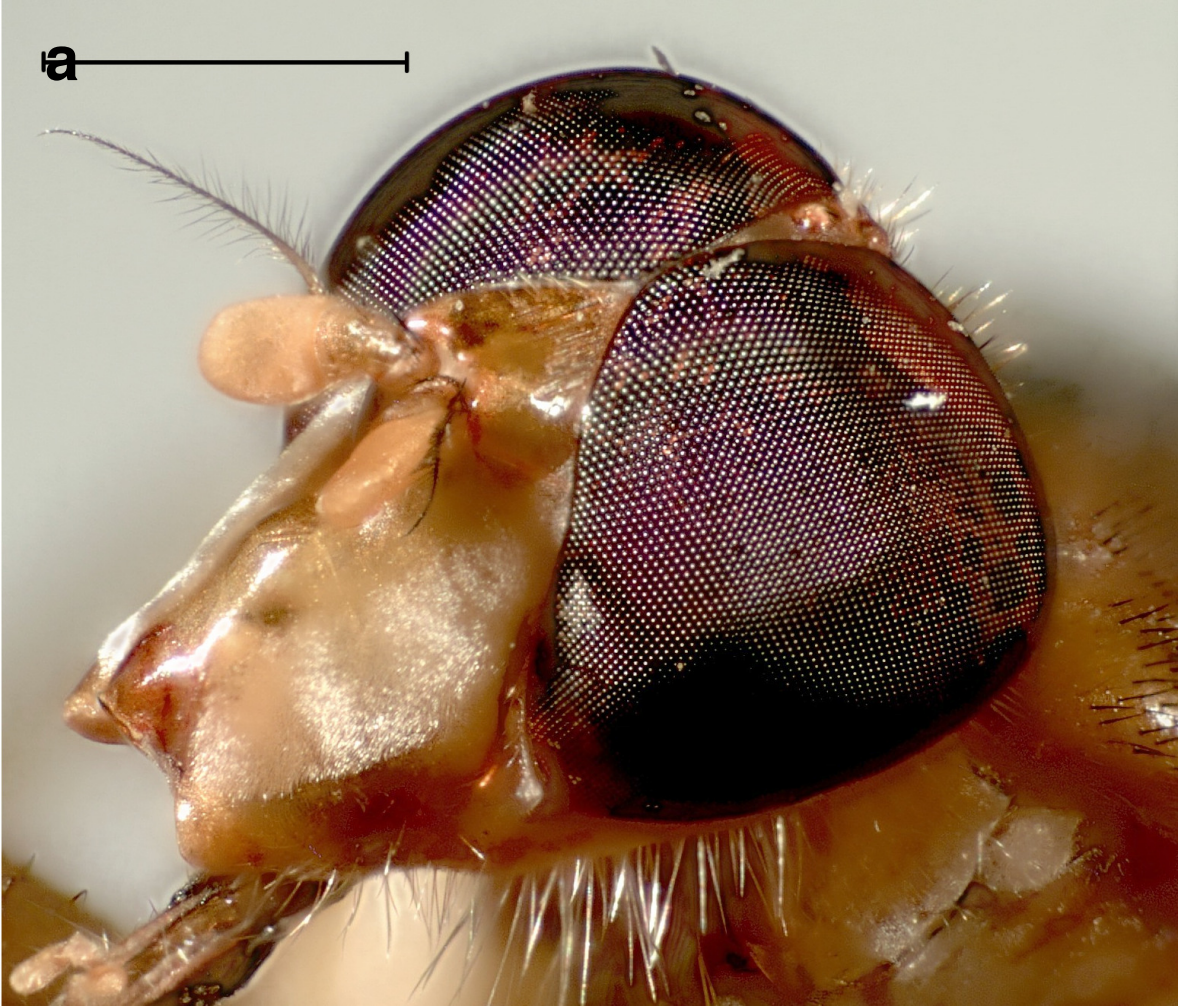
*H. ferruginea* Fallén.

**Figure 11b.**
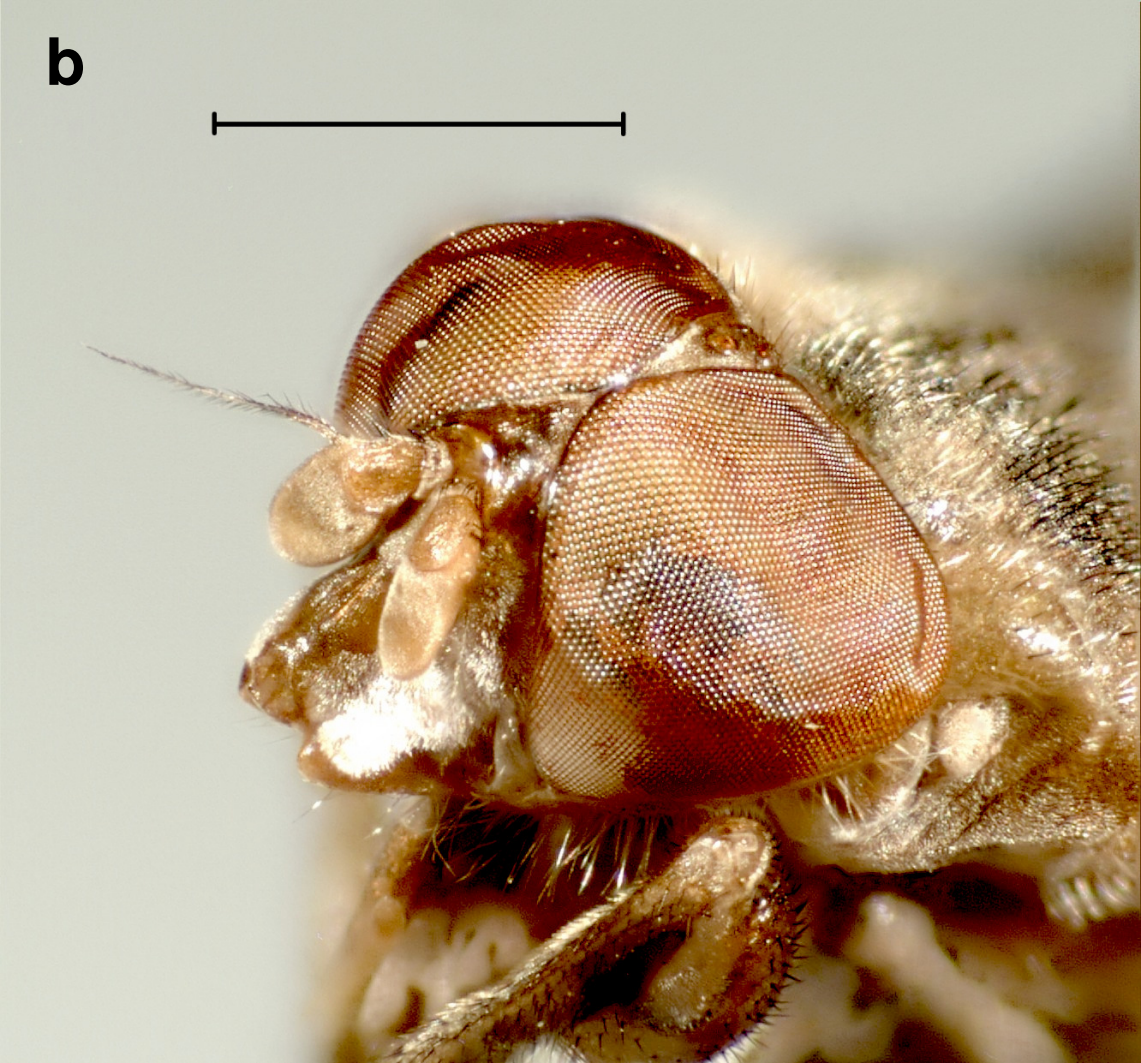
*H. ingrica* Stackelberg.

**Table 1.** Occurrence of remarkable Diptera species and wood-decaying macromycetes on fallen aspens in Kivach Nature Reserve (DBH - diameter at breast height; WB - windbreak; WT - windthrow).

	**A_1**	**A_2**	**A_3**	**A_4**	**A_5**	**A_6**	**A_7**	**A_8**	**A_9**	**A_10**
**Trunk parameters**										
The time since tree death	9	16	10	7	10	5	8	1	4	6
Decay class	1	1	2	2	1	1	2	1	1	1
DBH (cm)	41.4	54.1	49.7	70.4	78.7	36.9	29.9	33.8	27.4	74.5
Tree mortality mode	WB	WT	WB	WB	WB	WB	WT	WB	WT	WB
**Diptera (number of collected specimens)**										
*Atypophthalmus machidai* Alexander	0	0	0	0	2	0	0	0	0	0
*Discobola parvispinula* Alexander	0	0	0	0	0	0	1	0	0	0
*Gnophomyia acheron* Alexander	0	0	0	6	2	1	0	0	0	0
*Limonia badia* Walker	0	1	0	1	0	0	0	0	0	223
*Ectaetia subclavipes* Krivosheina	16	0	7	295	18	3	0	0	1	1
*Mesaxymyia kerteszi* Duda	1	0	0	0	0	0	0	0	0	0
*Impleta consorta* Plassmann	0	0	1	0	0	0	0	0	0	0
*Zygoneura bidens* Mamaev	15	0	0	0	0	0	0	0	0	1
*Agathomyia vernalis* Shatalkin	0	0	0	0	6	0	4	0	0	0
*Hammerschmidtia ingrica* Stackelberg	0	0	0	4	0	0	0	0	0	0
*Clusiodes tuomikoskii* Mamajev	26	0	0	0	0	0	0	0	0	2
**Fungi (presence/absence)**										
*Otidea onotica* (Pers.) Fuckel	0	0	1	0	0	0	0	0	0	0
*Peziza repanda* Pers.	0	0	1	0	1	0	0	0	0	1
*Scutellinia scutellata* (L.) Lambotte	0	1	0	0	1	0	0	0	0	1
*Pleurotus pulmonarius* (Fr.) Quél	0	0	0	0	1	0	0	0	1	0
*Armillaria cepistipes* Velen.	1	0	1	1	0	1	1	0	0	0
*Hericium cirrhatum* (Pers.) Nikol.	1	0	1	0	0	0	0	0	0	0
*Lentaria afflata* (Lagget) Corner	0	0	0	1	0	1	0	0	0	1
*Bjerkandera adusta* (Willd.: Fr.) P. Karst.	1	1	1	0	1	0	1	0	1	1
*Cerioporus leptocephalus* (Jacq.) Zmitr.	0	0	0	0	1	0	0	0	0	0
*Cerioporus squamosus* (Huds.) Quél.	0	0	1	0	0	0	0	0	0	0
*Fomes fomentarius* (L.: Fr.) Fr.	0	0	0	1	0	0	1	0	0	0
*Fomitopsis pinicola* (Sw.: Fr.) P. Karst	0	0	0	0	1	0	1	0	0	0
*Ganoderma applanatum* (Pers.) Pat.	0	1	0	0	0	0	0	0	0	0
*Hyphoderma setigerum* (Fr.: Fr.) Donk	0	0	0	1	0	0	0	0	0	1
*Inonotus obliquus* (Pes.: Fr.) Pilát	0	0	0	0	0	1	0	0	0	0
*Mycoacia fuscoatra* (Fr.) Donk	0	0	1	0	0	0	0	0	0	0
*Oxyporus corticola* (Fr.) Ryvarden	1	0	1	1	1	1	1	0	0	1
*Phellinus tremulae* (Bondartsev) Bondartsev et Borisov	1	1	1	0	1	0	1	1	1	1
*Royoporus badius* (Pers.) A.B. De	0	0	0	0	1	0	0	0	0	0
*Trametes ochracea* (Pers.) Gilb. et Ryvarden	1	0	1	1	1	0	0	0	0	1
*Trametes trogii* Berk.	0	0	0	0	1	0	0	0	0	0
*Xenasma rimicola* (P. Karst.) Donk	0	0	0	0	0	0	1	0	0	0
*Amphinema byssoides* (Pers.: Fr.) J. Erikss.	0	1	0	0	1	0	1	0	0	1
*Botryohypochnus isabellinus* (Fr.) J. Erikss.	0	0	0	0	0	1	1	0	0	0
*Chondrostereum purpureum* (Pers.: Fr.) Pouzar	1	0	0	0	0	0	0	0	1	1
*Coniophora arida* (Fr.) P. Karst.	0	0	0	0	0	1	0	0	0	0
*Coniophora olivacea* (Fr.: Fr.) P. Karst.	0	0	0	0	0	1	0	0	0	0
*Cylindrobasidium laeve* (Pers.) Chamuris	0	0	0	0	0	0	0	1	0	0
*Dichostereum boreale* Pouzar	0	0	1	0	0	0	0	0	0	0
*Kurtia argillacea* (Bres.) Karasiński	0	0	0	0	0	0	1	0	0	0
*Peniophora incarnata* (Pers.) P. Karst.	0	0	0	0	0	0	0	0	0	1
*Punctularia strigosozonata* (Schwein.) Talbot	0	0	0	0	1	0	0	0	0	0
*Resinicium bicolor* (Alb. et Schwein.: Fr.) Parmasto	0	0	0	0	0	0	1	0	0	0
*Scytinostroma galactinum* (Fr.) Donk	0	0	1	0	0	1	0	0	0	0
*Serpula himantioides* (Fr.) P. Karst.	0	0	1	0	0	0	0	0	0	0
*Subulicystidium longisporum* (Pat.) Parmasto	0	0	0	1	0	0	0	0	0	0
*Tomentella badia* (Link) Stalpers	0	0	0	0	0	0	1	0	0	0
*Tomentella bryophila* (Pers.) M. J. Larsen	0	1	1	0	0	0	1	0	0	0
*Tomentella cinerascens* (P. Karst.) Höhn. et Litsch	0	0	0	0	0	1	0	0	0	0
*Tomentella coerulea* (Bres.) Höhn. et Litsch.	0	1	0	0	0	0	0	0	0	0
*Tomentella crinalis* (Fr.) M. J. Larsen	0	0	0	0	0	0	1	0	0	0
*Tomentella lapida* (Pers.) Stalpers	0	0	0	0	0	0	1	0	0	0
*Tomentella stuposa* (Link) Stalpers	0	0	1	0	0	0	0	0	0	0
